# Instruction of College Students in Regard to Reproduction and Maternity*Prepared at the request of and read before the Public Health Education Committee of the Medical Society of the county of New York, at the New York Academy of Medicine, March 6, 1912.

**Published:** 1913-04

**Authors:** Elizabeth B. Thelberg

**Affiliations:** Poughkeepsie, N. Y.


### Instruction of College Students in Regard to Reproduction and Maternity.*

BY ELIZABETH B. THELBERG, M. D., POUGHKEEPSIE, N. Y.

Fifty years ago college education for women was a new and startling question; thirty years ago it was still a question; twenty years since the question was answered. Today it is a fact assured. Girls are going to college, and in increasing proportion, not the extraordinary or unusual girl, but the ordinary high school girl from nearly every city on the continent. The old tremors are ceasing to agitate society. The world has come to the clear and simple view of Matthew Vassar, expressed in one of his earliest communications to his board of trustees: "It occurred to me," said Mr. Vassar, "that woman, having received from her Creator the same intellectual constitution as man, has the same right as man to intellectual culture and development." Wellesley, Smith, Bryn Mawr, Barnard, the coeducational colleges and universities have justified his prescience. Cavilers have largely ceased from caviling and the occasional denunciations of the ill effects of the higher education of women come chiefly from specialists whose opportunities of observation have been chiefly among invalid women.

Leaving this almost threadbare subject, and taking the fact for granted that our American girls are going to college "not as single spies, but in battalions" how shall there be introduced into the college curriculum that instruction concerning their lives as women, as potential mothers without which they cannot rank as educated women?

All are not agreed as to the wisdom and necessity of such instruction. One eminent woman educator is quoted as saying that knowledge of these subjects and of hygiene in general is no more part of liberal college education than is dentistry. Now, though I bow in admiration before many of the decisions of this wise and lovely lady, here I take issue with her absolutely.

*Prepared at the request of and read before the Public Health Education Committee of the Medical Society of the county of New York, at the New York Academy of Medicine, March 6, 1912.

Indeed the matter to my mind does not need discussion, so obvious is it that the attitude is a mistaken one.

Are we to neglect so vital a matter—one of universal interest? Are we to miss so great an opportunity for putting this matter, so perverted often by the drama, the novel, the newspaper, even also by the pulpit, in its true light, scientifically and socially? A free and upright womanhood is not a blindfold womanhood.

Women should know the truth and the truth will make them free!

Furthermore, as in the case of children, if not taught these matters aright adolescence will teach them wrong, teach them with a blight over them, or with the veil of sentimentality and false emotion hindering all clear vision.

Who shall teach them? To my mind a physician one in whom they have that confidence engendered by successful practice. I believe that in the majority of cases the subject can be put with greatest moral earnestness by the physician in charge of the health of the college community.

Mr. Vassar, in this matter as in many others, was far ahead of his time in making his first appointments that of a resident physician who was at the same time professor of physiology and hygiene. Nevertheless, while the subject upon which I have been asked to speak this evening is one of great general public interest, that interest is for most people one of comparatively recent date. The period of my interest in it and of active teaching of the subject registers back for twenty-five years. It would not be profitable to take up the few minutes which are assigned to me in tracing the development, the gradual increase of subject matter covered in my own teaching. I will, therefore, give you a brief resume of its present status, premising only that more and more explicitness and frankness have been possible as the years have gone on and as the minds of parents as well as children have broadened toward the subject.

Outside my own department of physiology and hygiene, for the position includes still that of professor, as well as resident physician, a very large percentage of our young women enjoying admirable teaching in biology and zoology which obviously prepares the minds of such students for a much better grasp of the subject of advanced human physiology. The majority of these students elect the latter after having had the former, but the election is not limited to such students. In teaching human physiology I begin with the lowest forms of life and devote a lecture or two to the subject of evolution of the higher from the lower, teaching them in short to regard man, not as a fallen angel, but as a risen ameba. I then begin the demonstration by models, and by microscopic study of slides, with the reproductive organs of the female, trace the origin, growth, fertilization, and development of the ovum to the point of classification of tissues, then go on to the usual study of the various systems of the body. This gives a very desirable background to later lectures upon maternity.

In addition to the elective courses in physiology which run through the year, I also offer a course in public hygiene which is very largely elected. In this course I classify all the communicable diseases, teaching the essentials in relation to their history, transmission, and prophylaxis. In this classification I include gonorrhea and syphilis, and treat them precisely as I have treated the others. That is, I give a brief outline of the history of each disease, its prevalence, usual and unusual modes of communication, its chief symptoms, its possible complications and results. I give the history of our knowledge of the germ of each and its diagnostic importance. I lay great stress upon the hereditability of syphilis, and also upon the ultimate results of gonorrhea as a cause of sterility, of miscarriage, and of subsequent surgical procedures.

I tell my students plainly that no man leading a promiscuously licentious life ever escapes gonorrhea and rarely syphilis; that, however much of repentance he may bring to his marriage, he brings, as a rule, after such a life an infected body, and furthermore in the case of one of these diseases an infection which may transmit itself not only to an innocent wife, but to children as well.

It seems to me exceedingly important that the venereal diseases should be classified quite as a matter of course along with the other communicable diseases, and that the lectures upon them should be absolutely free from sensationalism and, above all, from any tinge of emotionalism or of sentimentalism, that lowest deep of all.

At the same time I think it very important that young girls should receive no exaggerated nor alarmist view in relation to the subject. I avoid figures and percentages, in short, pessimism.

Besides the two courses in physiology and sanitation which run throughout the year, I give a course of six lectures upon personal hygiene each Autumn to all new students. These numbers vary from 350 to 325.

In these lectures, in addition to the general subjects comprised in personal hygiene, I give one lecture upon the subjects of constipation and menstruation.

But my chief work in relation to our subject tonight is given toward the close of the senior year, when I offer to the graduating class a course of four lectures upon reproduction, maternity, and the care and feeding of young children. These lectures are usually attended, I think I may say, by every member of the senior class and I have had earnest assurances for many years that they have been of real service in the lives of many women.

Since some students may perhaps not have elected my other classes, I recapitulate briefly in these lectures the information in regard to the venereal diseases, dwelling especially in relation to them upon sterility and abortion.

I also speak very plainly upon the subject of criminal abortion, placing it where it belongs as a State prison offense, but giving the information necessary to an understanding of what a justifiable, i. e., a legal abortion, miscarriage, or premature birth is, stating that it should never be performed on the word of any one physician, but only after consultation with the best adviser possible to obtain.

I also devote considerable time to the consideration of heredity and eugenics, explain to them Mendel's law, show them several pedigree charts, and explain fully the disastrous results of combining bad, and especially similar bad strains of inheritance.

In these lectures I use models of the uterus and its appendages, of the ovary, and of the developing ovum up to about three months. I explain and show them carefully the development and the use of the afterbirth, and dwell upon the danger attending infection of the placental wound.

I also instruct them in regard to muscle tone of the uterine wall, and explain that the tone of that muscle corresponds to the muscle tone of the body as a whole and shares with the body muscles vigor and vitality, as well as laxness and flabbiness.

I endeavor to show how with the carriage of the child to full term, Nature is with the mother every step of the way, and that inconvenience, discomfort, and pain can be, by proper living, reduced far below the average apprehension; that even if not reduced they are not worthy of mention in comparison with the glorious privilege of being a mother. I instruct them briefly as to the cutting of the cord, the appearance of the afterbirth, and the immediately subsequent needs of the mother and the child, not forgetting the treatment of the child's eyes, etc.

In relation to the feeding of the child, I adjure them never to let mother-in-law or sister or female friend or trained nurse or physician debar them from the duty, pleasure and privilege of nursing their own babies should they be fortunate enough to have any.

Someone has asked me if I ever mention prenatal influence. I

do. I warn against silly tales, both spoken and printed, in relation to the subject, but at the same time I tell them that there is such a thing for both parents and that it begins with the first conscious choice of the individual between right and wrong, physical and moral.

These lectures are not easy to give. I feel as if they were at once the most important and hardest part of my year's work. A slight error in taste and judgment would nullify much of the good that one fain would do.

In my opinion no amount of information can compensate for a loss of the sense of decency. Above all things such lectures must not be touched with the taint of sentimentality. Neither should they be made purely, coldly, inhumanly scientific. The human touch, now and again a touch of humor, should lighten up the profound seriousness with which youth is so prone to take all its problems.

An exact and wide knowledge of the subject should be, obviously, the first essential in teaching. I have sometimes felt of late that with the growing demand for instruction upon these subjects this last essential was not always met in would be instructors, that sometimes "a little more—and how much it is"—than was necessary or wise was given.

Is it not possible that the pendulum may swing too far away from the old and harmful reticence, and the fastidious and critical mind of adolescence be offended by undue detail, and the few prurient minded be stimulated to unclean curiosity? It is the golden mean we are all seeking. How high and difficult of attainment it is, only those who try unceasingly to reach it know.—*N. Y. Med. Journal.*

---

It is a good thing to be rich, and a good thing to be strong, but it is a better thing to be beloved of many friends.—Euripides.

---

"Resolve to be thyself, and know that he
Who finds himself, loses his misery!"

---

"If I can stop one heart from breaking,
I shall not live in vain;
If I can ease one life that's aching,
Or cool one pain,
Or help one fainting robin into nest again,
I shall not live in vain."

